# A Note upon the Importance of Barium Salts

**Published:** 1909-01-30

**Authors:** 


### A NOTE UPON THE IMPORTANCE OF BARIUM SALTS.

It sometimes happens that a vegetable drug which for generations has been accredited with doing good in certain ailments is carefully analysed for an active principle; and when none such is found the drug in question is apt to be scientifically voted useless. The active principles usually sought are alkaloids, glucosides, or other allied organic compounds. Perhaps too little stress is laid upon the inorganic constituents and their actions. It is possible that a plant may contain no demonstrable alkaloid, glucoside, or other ordinary active principle, and yet be extremely active. This is brought out incidentally by some very interesting researches recorded by Dr. Crawford upon the question of the nature of "loco" disease.

This complaint occurs in animals upon certain ranches in America and Australia: the symptoms are partly gastro-intestinal and partly nervous, the end more or less rapid death. Post-mortem examination reveals nothing characteristic, and the whole trouble is popularly attributed to ingestion of the "loco" plant. The latter appears to derive its name from the disease it causes, for "loco" in Spanish signifies crazy, and applies to the behaviour of the poisoned animals, whose nervous disturbances seem to include delusions of space.

That the plant is really the cause of the fatal poisoning was shown experimentally. Dr. Crawford, who fed rabbits upon an aqueous extract of the plants, found that the animals would die within a very short time with acute symptoms of poisoning. Repeated small doses caused emaciation and death usually in ten days. The rabbits all showed peculiar psychical symptoms which closely resembled those of "locoed" animals. Abortion was also very common, as it is in "loco" disease.

All previous attempts to isolate an "active principle" from the plants had been unsuccessful. So far as laboratory investigations went, the plant appeared to be inert. When, however, the dried residue came to be carefully analysed for its inorganic constituents barium was found to be present in relatively very large amounts—76 to 173 milligrams of barium acetate in every 200 grams of dried plant.

Dr. Crawford next found that the feeding of rabbits upon repeated small doses of barium led to symptoms of acute or chronic poisoning like those of loco disease. Further, if the barium were removed from the preparations of loco plant and an animal were fed upon the barium-free product no harm befel it; whereas if the barium were now re-introduced the animal very shortly died.

It is not every loco plant that is thus rich in barium. Plants collected in some localities may be relatively free from barium, in which case animals can eat them with impunity. It seems obvious, therefore, that the occurrence of loco disease depends upon the prevalence of barium in the soil of the ranches over which the cattle graze, and that the metal is taken up by the plants which, upon ingestion, produce the toxic symptoms. Barium salts clearly deserve more attention than they have yet received in connection with human as well as animal and plant metabolism.

